# Exosomal TUBB3 mRNA expression of metastatic castration‐resistant prostate cancer patients: Association with patient outcome under abiraterone

**DOI:** 10.1002/cam4.4168

**Published:** 2021-07-28

**Authors:** Sha Zhu, Yuchao Ni, Guangxi Sun, Zilin Wang, Junru Chen, Xingming Zhang, Jinge Zhao, Xudong Zhu, Jindong Dai, Zhenhua Liu, Jiayu Liang, Haoran Zhang, Yaowen Zhang, Pengfei Shen, Hao Zeng

**Affiliations:** ^1^ Department of Urology Institute of Urology West China Hospital Sichuan University Chengdu Sichuan China

**Keywords:** abiraterone acetate, exosomes, messenger, prostatic neoplasms, RNA, *TUBB3*

## Abstract

**Background:**

To use ddPCR to quantify plasma exosomal class III β‐tubulin (βIII‐tubulin, *TUBB3,* encoded by the *TUBB3* gene) mRNA expression in metastatic castration‐resistant prostate cancer (mCRPC) patients, and study the association of this expression with abiraterone efficacy.

**Methods:**

Blood samples were prospectively collected from 52 mCRPC patients using abiraterone as first‐line therapy to measure plasma exosomal *TUBB3* mRNA expression value before the initiation of abiraterone. Study endpoints were PSA response rate, PSA‐progression‐free survival (PSA‐PFS), and overall survival (OS, from CRPC to death).

**Results:**

Patients with positive exosomal *TUBB3* expression showed shorter PSA‐PFS (negative *TUBB3* vs. positive *TUBB3*: 11.0 vs. 7.9 months; *p* = 0.014). Further analysis demonstrated that patients with strongly positive exosomal *TUBB3* (>20 copies/20 µl) was associated with even shorter PSA‐PFS (negative *TUBB3* vs. positive *TUBB3* [<20 copies/20 µl] vs. strongly positive *TUBB3* [>20 copies/20 µl]: 11.0 vs. 8.3 vs. 3.6 months, *p* = 0.005). In multivariate analyzes, *TUBB3* (+) (HR: 2.114, *p* = 0.033) and ECOG score >2 (HR: 3.039, *p* = 0.006) were independent prognosticators of poor PSA‐PFS. PSA response and OS did not present significant differences.

**Conclusion:**

The exosomal *TUBB3* mRNA expression level is associated with poor PSA‐PFS of abiraterone in mCRPC patients. The detection of exosomal *TUBB3* can be valuable in their management.

## Graphical abstract

In this study, we presented this novel, non‐invasive biomarker for abiraterone resistance in mCRPC patients using a ddPCR approach to quantificationally measure the exosomal TUBB3 mRNA expression. We found that a higher TUBB3 level is correlated with shorter progression time after first‐line abiraterone treatment in mCRPC patients.

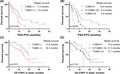

**Lay summary**

Metastatic castration‐resistant prostate cancer (mCRPC) is the end stage of prostate cancer with high mortality. Although recent years have seen the satisfying efficacy of abiraterone for mCRPC patients, a certain portion of people still responds poorly to abiraterone. Currently, there are a lack of potential easy, cheap, and noninvasive biomarkers to predict patients' response to abiraterone. This study found that a blood‐based test of exosomal *TUBB3* can effectively identify candidates who are unlikely to respond to abiraterone therapy. With this information, the patients and their physicians can be better informed to make reasonable and evidence‐based treatment plans.

## 1 INTRODUCTION

Microtubules are the most studied cytoskeleton components, which actively participate in extensive crosstalk in the tumor microenvironment.^1^ Class III β‐tubulins (βIII‐tubulin, encoded by the *TUBB3*, Class III β‐tubulin (βIII‐tubulin) gene) are a dynamic constitute of microtubules, prominently confined to neural tissues and testis. βIII‐tubulins play an essential role in neural development; therefore, it can be considered as a pan‐neuronal marker for early detection of neural cell differentiation.^2^ Nevertheless, the clinical significance of βIII‐tubulin is more than tissue‐specify. Studies also show that βIII‐tubulin level arises in hypoxia and hypoglycemia conditions,^3^ implying its potential in tumor initiation. Indeed, the expression of βIII‐tubulin is not uncommon in a variety of solid tumors.^1^, ^4^

βIII‐Tubulin serves as a biomarker not only for tumorigenesis, but also for tumor aggressiveness and metastasis.^5^, ^6^, ^7^ Previous studies have mainly researched βIII‐tubulin's indication of resistance to chemotherapeutic drugs. Due to its intrinsic characteristics as microtubule components, βIII‐Tubulin was believed to directly link with resistance to microtubule‐target drugs in many tumors, including prostate cancer.^6^, ^8^, ^9^ However, reports are contradicting to this proposition,^10^, ^11^ and evidence suggests that βIII‐tubulin is actually more than a pure predictive marker of taxanes; it also participates in the sensitivity for other forms of drugs,^12^ indicating a broader role for this β‐tubulin isotype in tumor biology. In other words, the function of *TUBB3* can be more than merely constituting microtubules. Currently, more evidence is in favor of another more complex network in which βIII‐tubulin is a component of a prosurvival signal pathway in response to the hostile microenvironment.

Prostate cancer is largely driven by androgen receptor (AR) signaling activation. The last decade has seen the application and the consequent success of next‐generation AR signaling inhibitors. Ultimately, however, patients will still succumb to this final stage of the continuum of prostate cancer. The expression of βIII‐tubulin is increased in metastatic castration‐resistant prostate cancer (mCRPC).^13^ Previous reports have suggested that AR was correlated with the upregulation of *TUBB3* expression in colorectal cancer,^14^ and activated AR signaling could enhance tubulin expression.^14^, ^15^ Therefore, βIII‐tubulin might also affect the resistance against the next‐generation AR targeting drugs like abiraterone in mCRPC. However, this association has not been studied yet. The lack of simple, noninvasive, and reliable detection methods of the *TUBB3* level are the significant limitations for understanding the potential predictive and prognosis value of *TUBB3* in prostate cancer. Since it is not always easy to obtain repeated biopsy samples in the clinical setting, tissue‐based detection is not feasible. In this case, plasma‐based exosomal *TUBB3* quantification seems promising in the extensive screening of patients preparing to use abiraterone. This study aimed to find out if an exosomal *TUBB3* expression could predict the efficacy of abiraterone and even overall survival (OS) in mCRPC patients.

## 2 PATIENTS AND METHODS

### 2.1 Study design and ethics

This is a prospective study using digital droplet polymerase chain reaction (ddPCR) to measure the plasma exosomal *TUBB3* mRNA expression value of mCRPC patients prior to their initiation of abiraterone. Our experiment protocols referred to a published study on *European Urology*.^16^ This study protocol was approved by the Medical Ethics Committee of West China Hospital, Sichuan University. Relevant detail has been explained to the patient himself and/or his caregivers. Consents for publication were obtained from the patients involved in this study. The study was performed under the Declaration of Helsinki.

### 2.2 Patient population

Patient inclusion criteria included: pathological diagnosis of prostate adenocarcinoma, mCRPC (serum testosterone <50 ng/dl; three consecutive rises in PSA 1 week apart over the nadir [>2 ng/ml] or radiological progression, according to EAU and PCWG3 guidelines), ready to use abiraterone acetate monotherapy, with complete pathological results in our institute, good patient compliance and able to complete the follow‐up regularly. Patients with prostatic ductal adenocarcinoma, small‐cell/large‐cell neuroendocrine prostate cancer (NEPC) were excluded.

Our team screened and chose eligible study participants in the outpatient department. Three milliliters of blood specimens was collected from each enrolled patient before the initiation of abiraterone treatment. A total of 52 mCRPC patients were enrolled in this study. Thereafter, clinical data, including patient baseline assessments, prior systemic treatments and responses, metastasis status and site, time of mCRPC diagnosis, PSA, blood routine examination, liver and kidney functions, computed tomography, bone scan, biopsy histology, and immunohistochemical (IHC) results were recorded. Patients were allowed to choose suitable second and multiline therapies if they progressed on abiraterone to maximize patient benefits. All patients were followed till death or the end of this study.

### 2.3 Extraction of 22Rv1 TUBB3 mRNA and its detection by digital ddPCR

We chose the 22Rv1 cell line to set up the ddPCR system since our preliminary experimental results showed that 22Rv1 carried a higher expression of *TUBB3* than C4‐2 cells (Figure S1). We extracted exosomes from the culture supernatant of 22Rv1 cells, and mRNA from exosomes was purified with an exoRNeasy Kit (QIAGEN) according to the provided manual. mRNA was reverse transcribed using the QuantiTect Reverse Transcription Kit (QIAGEN) according to the manufacturer’s instructions. The primers for exosomal *TUBB3* mRNA amplification were designed using Bio‐Rad SsoFast EvaGreen Supermix, and synthesized in our laboratory (*TUBB3*: forward, ATCAGCAAGGTGCGTGAGGAG; and reverse, TCGTTGTCGATGCAGTAGGTC). The reaction system comprised 20 μl of 10 µl 2× SsoFast EvaGreen Supermix, 3 µl cDNA template, 1 µl of each forward and reverse primer and 5 µl *water* to the total volume of 20 μl and assembled into individual wells. We added 70 µl droplet generation oil and placed the eight‐well cartridge into the droplet generator. Then, we transferred 40 µl of the droplet solution into a 96‐well PCR plate. The following conditions were used for the reverse transcriptase PCR reaction: 50℃ × 60 min, 95℃ × 10 min, 95℃ × 30 s, and 55℃ × 60 s (40 cycles), 98℃ × 10 min, 4℃ hold. We used the droplet reader for fluorescence signal quantification. The QuantaSoft software (Bio‐Rad) was used to measure the number of positive versus negative droplets for both fluorophores (FAM/HEX), and their ratio is then applied to a Poisson distribution to define the copy number of the target molecule, as copies per 20 microliters (copies/20 μl), in the input reaction. Exosomes from the culture supernatant of 22Rv1 cells were used as the positive control for further detection in patient blood samples. Actin was used as an internal control (forward, CCCAGCCATGTACGTTGCTA; and reverse, AGGGCATACCCCTCGTAGATG).

### 2.4 mRNA extraction from exosomes

Samples were all thawed and spun at 1500 *g* for 15 min to remove residual cellular debris before use. The plasma was then preserved at −80℃. We performed the plasma exosome isolation with the exoEasy spin columns of an exoRNeasy kit (Qiagen). The validation of the exosomes extracted from cells and blood samples by both exosomal markers western blotting and Nanoparticle Tracking Analysis is shown in Figure S2. We extracted mRNA from the vesicles by the QIAzol phenol/guanidine‐based lysis solution, added chloroform to QIAzol‐samples, and centrifuged at 12,000 *g* for 15 min at 4℃. Then we recovered the aqueous phase containing mRNA and applied to the RNeasy MinElute spin column, and abandoned the mRNA binding to contaminants. The remaining mRNA was eluted in 20 µl of the elution buffer. The RNAs were analyzed via the RNA6000 Nano assay (Agilent) for the determination of an RNA integrity number (RIN), and only analytes with a RIN ≥7.0 were included in this study.

### 2.5 Analysis of TUBB3 on plasma‐derived exosomal mRNA

*TUBB3* mRNA was analyzed by ddPCR with the One‐Step RT‐ddPCR kit, as described before.^16^ 22Rv1 mRNA was used as the positive control. Results were reported as copies of the *TUBB3* mRNA per 20 µl. Procedures for every patient sample were repeated for ≥2 times.

### 2.6 Outcome evaluation

The primary study endpoint was PSA‐progression‐free survival (PSA‐PFS) in this study. PSA‐PFS was defined as the time from the start of abiraterone therapy to progression according to PCWG3 consensus. The second study endpoint was OS, defined as CRPC diagnosis to all‐cause death. PSA response, defined as ≥50% decline in PSA level and maintenance for ≥4 weeks, was also analyzed.

### 2.7 Statistics

The ddPCR QuantaSoft software was used to calculate the absolute target concentration as copies/20 μl in the plasma. We used the chi‐squared test to compare the baseline characteristics between patients among different *TUBB3* expression groups. The Kaplan–Meier curve and log‐rank test were applied to compare the PSA‐PFS of patients in different groups. Cox regression was used in the univariate and multivariate analyzes of PSA‐PFS. Variables were all included in the multivariate analyzes using the forward selection (likelihood ratio) method. Data analyses were conducted with SPSS (version 25.0, IBM Corp.) and GraphPad Prism (version 8.0.2, GraphPad Software). Multivariate analyses were performed based on variables with *p* < 0.010 in univariate analyses by stepwise forward regression based on maximum likelihood estimation. All tests were two‐sided. A value of *p* < 0.050 was considered statistically significant.

## 3 RESULTS

### 3.1 Patient baseline characteristics

Between 2015 and 2019, 52 patients participated in this study and provided their blood samples at the time of mCRPC diagnosis. The median age of the patients was 70 years (IQR: 65–72), and the majority of patients (36/52, 69.2%) had an ECOG performance status of 0. All patients received maximum androgen blockade (bicalutamide plus LHRH agonist or orchiectomy) as initial treatment during their hormone‐sensitive stage, then abiraterone acetate plus prednisone (abiraterone: 1000 mg per day, prednisone: 10 mg per day) were treated as first‐line therapy after diagnosis of CRPC. The median follow‐up time of the whole cohort was 33.3 months. Median castration resistance‐free survival (CFS) and OS for this mCRPC cohort were 12.5 months (95% CI: 9.1–15.9) and 32.9 months (95% CI: 26.8–38.9). Initial PSA response (defined as a PSA decline>50%) rate after abiraterone treatment of the whole cohort was 48.1% (25/52) and 80.7% (42/52) patients had PSA progression during the follow‐up time, with median PSA‐PFS being 8.6 months (95% CI: 8.0–9.3). In addition, 48.1% (25/52) died during the follow‐up period.

### 3.2 *TUBB3* detection and baseline summary

Exosomal *TUBB3* mRNA positive rate was 42.3% (22/52). To be more specific, the number of patients for negative, positive (<20 copies/20 µl), and strongly positive (>20 copies/20µl) were 22/52 (42.3%), 27/52 (51.9%), and 3/52 (5.8%), respectively. Baseline characteristics were well balanced between the *TUBB3* (−) and *TUBB3* (+) expression groups. A widely used pan‐neuronal marker notwithstanding, *TUBB3* expression was not related to neuroendocrine differentiation (NED, defined as positivity of generic NE markers chromogranin A and synaptophysin) in conventional prostate adenocarcinoma (*p* = 0.653). No difference was found in CFS between the two groups (*p* = 0.545). Detailed information is shown in Table 1.

**TABLE 1:** Baseline characteristics

	Total cohort (*n* = 52)	TUBB3 (−) (*n* = 22)	TUBB3 (+) (*n* = 30)	*p* value
Age (y)				
≤70, *n* (%)	27 (51.9)	12 (54.5)	15 (50.0)	0.966
>70, *n* (%)	25 (48.0)	10 (45.4)	15 (50.0)	
Baseline PSA (ng/ml)				
<100, *n* (%)	15 (28.8)	8 (36.3)	7 (23.3)	0.475
≥100, *n* (%)	37 (71.1)	14 (63.6)	23 (76.6)	
Visceral metastases				
Negative, *n* (%)	49 (94.2)	20 (90.9)	29 (96.6)	0.781
Positive, *n* (%)	3 (5.7)	2 (9.0)	1 (3.3)	
Baseline LDH (IU/L)				
≤300, *n* (%)	46 (88.4)	22 (100.0)	24 (80.0)	0.073
>300, *n* (%)	6 (11.5)	0	6 (20.0)	
Baseline ALP (IU/L)				
≤160, *n* (%)	33 (63.4)	15 (68.1)	18 (60.0)	0.754
>160, *n* (%)	19 (36.5)	7 (31.8)	12 (40.0)	
Baseline HGB (g/L)				
≤120, *n* (%)	10 (19.2)	3 (13.6)	7 (23.3)	0.603
>120, *n* (%)	42 (80.7)	19 (86.3)	23 (76.6)	
ECOG PS				
<2, *n* (%)	43 (82.6)	20 (90.9)	23 (76.6)	0.332
≥2, *n* (%)	9 (17.3)	2 (9.0)	7 (23.3)	
CFS (months)				
≤12, *n* (%)	25 (48.0)	9 (40.9)	16 (53.3)	0.545
>12, *n* (%)	27 (51.9)	13 (59.0)	14 (46.6)	
Other treatment after ABI				
Negative, *n* (%)	25 (48.0)	9 (40.9)	16 (53.3)	0.545
Positive, *n* (%)	27 (51.9)	13 (59.0)	14 (46.6)	
Pre‐treatment PSA (ng/ml)				
≤100, *n* (%)	40 (76.9)	20 (90.9)	20 (66.6)	0.086
>100, *n* (%)	12 (23.0)	2 (9.0)	10 (33.3)	
ISUP/WHO group				
≤4, *n* (%)	10 (19.2)	3 (13.6)	7 (23.3)	0.603
>4, *n* (%)	42 (80.7)	19 (86.3)	23 (76.6)	
NED				
Negative, *n* (%)	41 (78.8)	18 (81.8)	23 (76.7)	0.653
Positive, *n* (%)	11 (21.2)	4 (18.2)	7 (23.3)	

Abbreviations: ABI, abiraterone; ALP, alkaline phosphatase; CFS, castration‐resistance free survival; ECOG PS, Eastern Cooperation Oncology Group performance status; HGB, hemoglobin; LDH, lactate dehydrogenase; NED, neuroendocrine differentiation; PSA, prostate‐specific antigen; TUBB3, class III β‐tubulin.

### 3.3 Prognostic value of exosomal *TUBB3* mRNA expression

**FIGURE 1:**
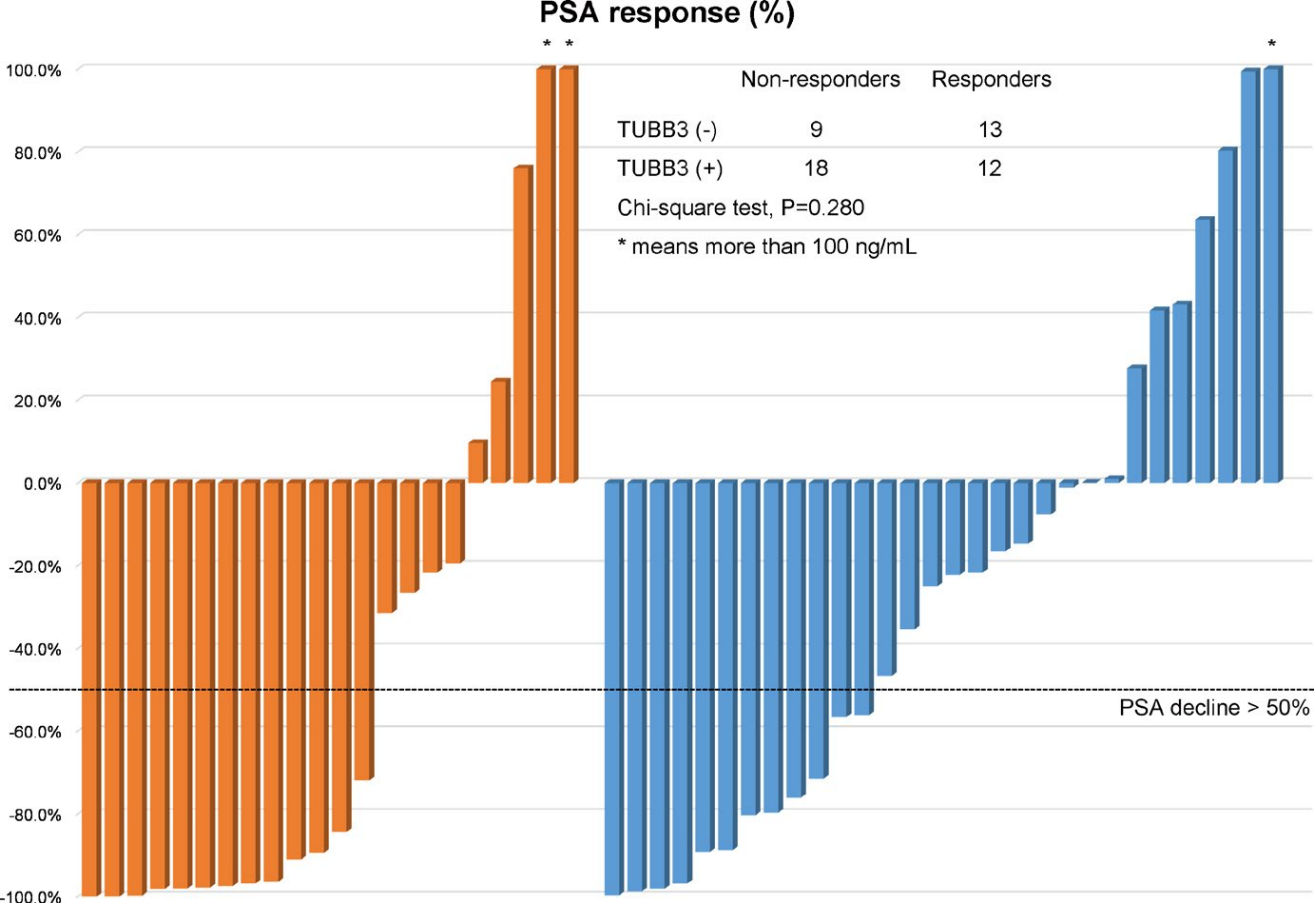
Waterfall Plot showing the best PSA change of patients and *TUBB3* status. The dotted line illustrates the threshold for PSA response definition (≥50% reduction in PSA serum level from baseline). Asterisks depict an increase of >100% in best PSA change. *TUBB3*, class III β‐tubulin; PSA, prostate‐specific antigen

**FIGURE 2:**
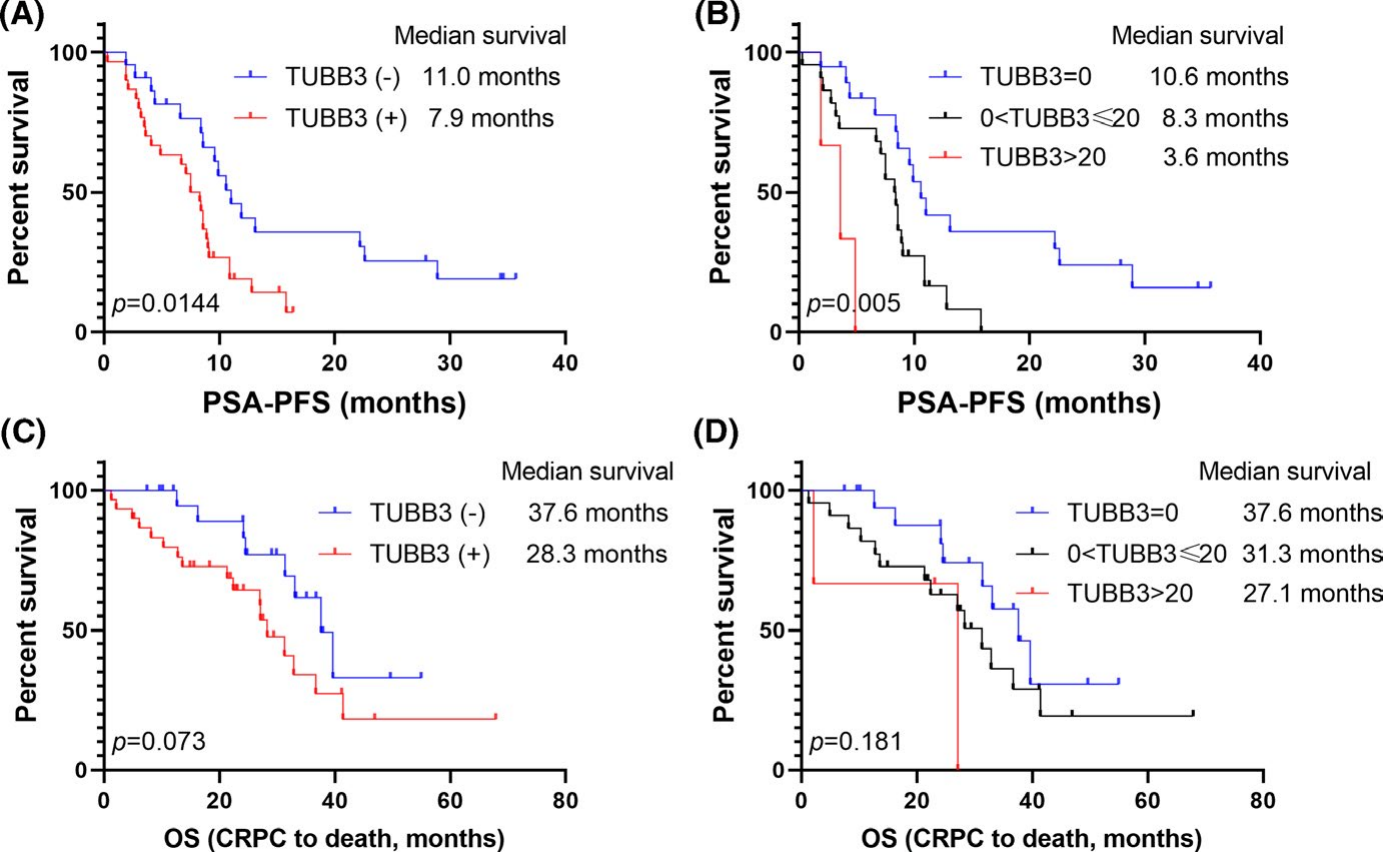
Kaplan–Meier curves of PSA‐PFS for: (a) patients with positive and negative *TUBB3* expression; B, negative, positive (≤20 copies/20 µl), strongly positive (>20 copies/20 µl) patients. *TUBB3* = class III β‐tubulin

*TUBB3* expression had no significant influence on the initial PSA response of the patients treated with abiraterone [13/22, 59.1% in *TUBB3* (−) group, 12/30, 40% in *TUBB3* (+) group, chi‐square tests, *p* = 0.280] (Figure 1). On account of PFS, the *TUBB3* (+) group showed a significantly shorter median PSA‐PFS (7.9‐Mo vs. 11.0‐Mo, *p* = 0.014) (Figure 2). PSA‐PFS also decreased as the positivity got stronger (median PSA‐PFS: *TUBB3* negative group: 10.6‐Mo; *TUBB3* positive group: 8.3‐Mo; *TUBB3* strongly positive group: 3.6‐Mo; *p* = 0.005) (Figure 2). Univariate survival analyzes revealed that *TUBB3* (+) (*p* = 0.018), ECOG≥2 (*p* = 0.018), and pretreatment PSA ≥100 ng/ml (*p* = 0.038) were associated with shorter PSA‐PFS. Multivariate analyses results suggested that ECOG ≥2 (HR: 3.039, 95% CI: 1.378, 6.699, *p* = 0.006) and *TUBB3* (+) (HR: 2.114, 95% CI: 1.063, 4.203, *p* = 0.033) were independent prognosticators of poor PSA‐PFS after abiraterone treatment (Table 2). However, *TUBB3* (+) did not predict OS (*p* = 0.079) (Table 3, Figure 2).

**TABLE 2:** Univariate and multivariate analyzes of PSA‐PFS

	Univariate analyzes		Multivariate analyzes	
	HR (lower limit, upper limit)	*p* value	HR (lower limit, upper limit)	*p* value
TUBB3 (+)	2.271 (1.152, 4.475)	0.018	2.114 (1.063, 4.203)	0.033
Age >70	0.864 (0.464, 1.609)	0.645		0.822
Baseline PSA >100 ng/ml	1.339 (0.681, 2.629)	0.397		0.283
Visceral metastasis	1.383 (0.424, 4.509)	0.590		0.213
LDH >300 IU/L	1.140 (0.404, 3.214)	0.805		0.696
ALP >160 IU/L	0.834 (0.442, 1.574)	0.576		0.441
HB>120 g/L	0.495 (0.230, 1.063)	0.071		0.186
ECOG PS ≥2	3.352 (1.535, 7.319)	0.002	3.039 (1.378, 6.699)	0.006
CFS >12 months	0.643 (0.350, 1.184)	0.156		0.231
PSA before ABI>100 ng/ml	2.210 (1.045, 4.673)	0.038		0.297
ISUP/WHO group>4	0.943 (0.434, 2.051)	0.883		0.703

Abbreviations: ABI, abiraterone; ALP, alkaline phosphatase; CFS, castration‐resistance free survival; ECOG PS, Eastern Cooperation Oncology Group performance status; HGB, hemoglobin; HR, hazard ratio; LDH, lactate dehydrogenase; PSA, prostate‐specific antigen; TUBB3, class III β‐tubulin.

**TABLE 3:** Univariate and multivariate analyzes of OS

	Univariate analyzes		Multivariate analyzes	
	HR (lower limit, upper limit)	*p* value	HR (lower limit, upper limit)	*p* value
TUBB3 (+)	2.137 (0.915, 4.989)	0.079		0.283
Age >70	0.960 (0.434, 2.121)	0.919		0.942
Baseline PSA >100 ng/ml	0.847 (0.360, 1.991)	0.703		0.613
Visceral metastasis	3.157 (0.904, 11.025)	0.072	4.277 (1.167, 15.677)	0.028
LDH >300 IU/L	2.111 (0.603, 7.393)	0.243		0.416
ALP >160 IU/L	0.758 (0.330, 1.742)	0.514		0.551
HB >120 g/L	0.611 (0.252, 1.479)	0.275		0.479
ECOG PS ≥2	2.586 (1.020, 6.558)	0.045		0.094
CFS >12 months	0.538 (0.243, 1.194)	0.128		0.392
Other treatment after ABI	0.615 (0.280, 1.352)	0.226		0.260
PSA before ABI >100 ng/ml	2.476 (1.059, 5.787)	0.036	2.901 (1.204, 6.993)	0.018
ISUP/WHO group >4	0.932 (0.350, 2.487)	0.889		0.703

Abbreviations: ABI, abiraterone; ALP, alkaline phosphatase; CFS, castration‐resistance free survival; ECOG PS, Eastern Cooperation Oncology Group performance status; HGB, hemoglobin; HR, hazard ratio; LDH, lactate dehydrogenase; PSA, prostate‐specific antigen; TUBB3, class III β‐tubulin.

## 4 DISCUSSION

This study presented this novel, noninvasive biomarker for abiraterone resistance in mCRPC patients using a ddPCR approach to quantificationally measure the exosomal *TUBB3* mRNA expression. We found that a higher *TUBB3* level is correlated with shorter progression time after first‐line abiraterone treatment in mCRPC patients, although it did not transfer into significant OS benefit probably due to the confounding multiline therapies.

We suppose plasma‐based exosomal detection for *TUBB3* mRNA is a reliable approach. For decades, tissue‐derived biomaterial has been the cornerstone of comprehensive cancer management, while it has some obviously inherent drawbacks. Subsequent developed liquid biopsy methods are just to make up for these unfeasible occasions, such as invasive surgical procedures, tumor heterogeneity, tumor progression, and dynamic treatment monitoring. Studies have demonstrated the value of blood‐based biomarkers of liquid biopsy in prostate cancer, especially advanced ones.^17^ Exosome‐based approaches, to be specific, have their unique advantage as taking our perspective into the subcellular level. This method of quantifying exosomal *TUBB3* expression based on mRNA circulating in plasma is more sensitive than circulating tumor cells and has a lower false‐negative rate. Also, exosomes are more stable and able to fuse with cell membranes; therefore, they have potential in targeted drug development.^18^ As this plasma‐derived approach does not rely on tumor biopsy tissues, it provides opportunities for dynamically monitoring for changes in tumor molecular features over time, which is rather necessary for the stage of mCRPC due to the interpersonal heterogeneity.

The observation that exosomal *TUBB3* being a potential noninvasive marker to identify candidates who are unlikely to respond to abiraterone therapy is novel and with little parallel data to compare with. We have several propositions to this phenomenon. First, βIII‐tubulin possibly acts as a pressure response mediator promoting tumor progression and/or abiraterone resistance in a hostile environment. Studies suggest that long‐term androgen deprivation therapy under hypoxic conditions precipitates adaptive AR‐independence.^19^ Besides, *TUBB3* is also considered an angiogenic marker.^20^ Although hypoxia and angiogenesis are long‐standing tumorigenic factors, studies on these factors have not been very translational in prostate cancer, probably owning to this disease's marked genetic and molecular heterogeneity. We hypothesize that, in *TUBB3*‐expressing mCRPC, hypoxia and angiogenesis may increase the prosurvival signals that βIII‐tubulin provided for tumor cells, which is beyond the mere scope of microtubule dynamics.^21^

Also, βIII‐tubulin could be interpreted as an indication of stemness, a more “dedifferentiated” or “progenitor‐like” phenotype in non‐neural tumors.^22^ While βIII‐tubulin acts in mature neurons and neural tumors as recapitulating its process in embryonic neurogenesis,^23^ this protein is not neuron‐specific and expressed in many non‐neural tumors. *TUBB3* gene is one of the targeted genes of RE‐1 silencing transcription factor (REST).^24^ Thus, *TUBB3* overexpression may be linked to the dysfunction of REST, which can lead to stemness and cellular plasticity when combined with AR‐antagonists.^25^ In other words, the prolonged therapeutic use of AR‐antagonists as a selective pressure could induce tumor subclones with higher malignancy, especially in the case of mCRPC. Moreover, the fact that CFS was not statistically different between the *TUBB3*‐positive and ‐negative groups also suggests this impact being time dependent. Although these fore‐mentioned mechanisms are more extensively studied in NEPC, our data showed no correlation between *TUBB3* expression and NED in conventional prostate adenocarcinoma, consistent with studies in neuroendocrine lung cancers.^26^

This study has multiple limitations. First, the number of registered patients is small. Second, all patients are from a single center, and thus selection bias cannot be ruled out. In order to obtain a more in‐depth understanding of the progression and drug resistance mechanism of mCRPC patients, future studies can focus on comparing *TUBB3* expression in different disease stages, and dynamic monitoring is even more recommended. Besides, *TUBB3* expression among diverse histological subtypes of prostate cancer is also worth investigating.

## 5 CONCLUSION

The rapid advance of pharmacogenomics and targeted medicine has aroused scientific interest in biomarkers, combined with the fact that nearly all prostate cancer patients will develop inevitable resistance to AR‐targeted therapies; therefore, identifying patients with shorter response time is particularly germane for timely treatment replacement. As suggested in previous studies, the heterogeneity and plasticity of the molecular nature of prostate cancer need to be justified by the combination of biomarkers instead of single markers or markers with very similar targets for monitoring the treatment course.^27^ Besides those widely recognized biomarkers, *TUBB3* is likely to participate in a more intricate and extensive network within the prostate cancer progression and drug resistance mechanism. In conclusion, the exosomal *TUBB3* mRNA expression level is associated with abiraterone efficacy in mCRPC patients. The noninvasive detection of *TUBB3* can be potential in the management of mCRPC patients under abiraterone.

## CONFLICT OF INTEREST

The authors declare no conflict of interest.

## Supporting information

Figure S1

Figure S2

## Data Availability

Research data are not shared due to patient privacy.
