# Supplementary figures and images for: Exosomal TUBB3 mRNA expression of metastatic castration‐resistant prostate cancer patients: Association with patient outcome under abiraterone
Source: Cancer Med. 2021 Jul 28;10(18):6282–90. doi: 10.1002/cam4.4168 (PMC8446399; doi:10.1002/cam4.4168)

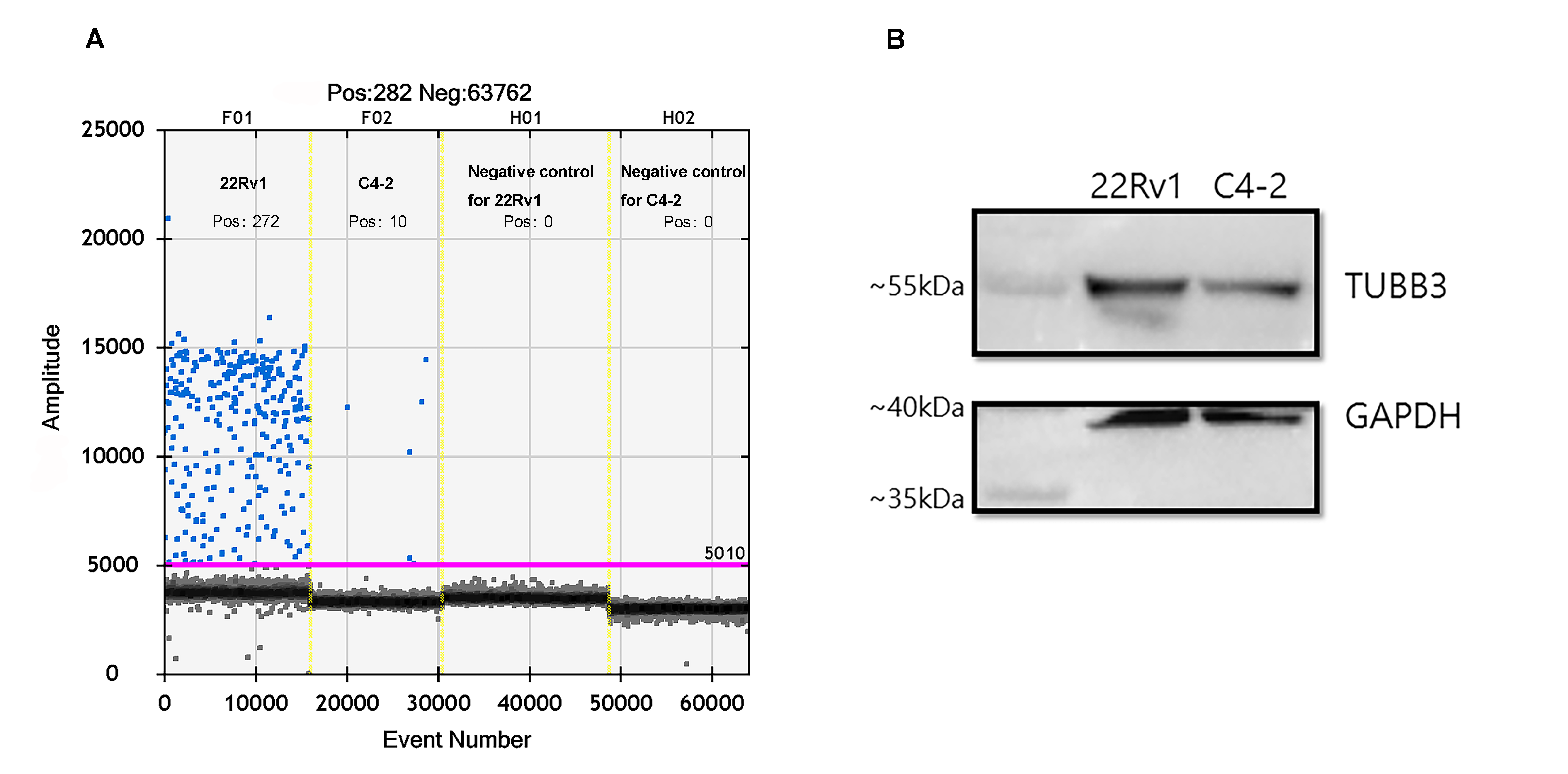

Supplement: Supplementary file 1 — Figure S1 [file CAM4-10-6282-s002.tif]

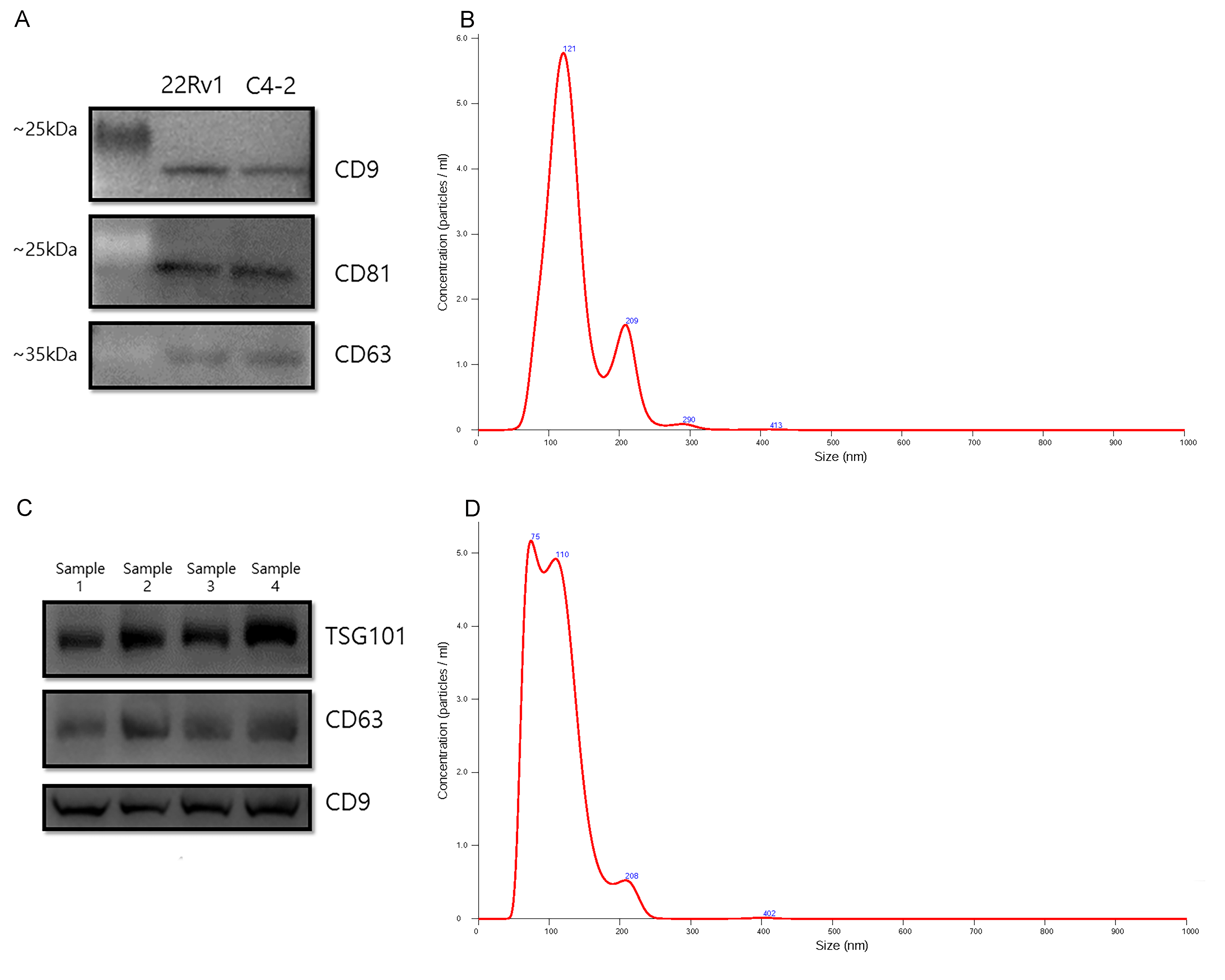

Supplement: Supplementary file 2 — Figure S2 [file CAM4-10-6282-s001.tif]
